# Development of Flame-Retarded Nanocomposites from Recycled PET Bottles for the Electronics Industry

**DOI:** 10.3390/polym11020233

**Published:** 2019-02-01

**Authors:** Ferenc Ronkay, Béla Molnár, Ferenc Szalay, Dóra Nagy, Brigitta Bodzay, István E. Sajó, Katalin Bocz

**Affiliations:** 1 Department of Polymer Engineering, Faculty of Mechanical Engineering, Budapest University of Technology and Economics, Műegyetem rkp. 3, H-1111 Budapest, Hungary; ronkay@pt.bme.hu (F.R.); molnarb@pt.bme.hu (B.M.); ferencszalay1@gmail.com (F.S.); n.dora9@gmail.com (D.N.); 2 Imsys Ltd., Material Testing Laboratory, Mozaik Street 14/A., H-1033 Budapest, Hungary; 3 Department of Organic Chemistry and Technology, Faculty of Chemical Technology and Biotechnology, Budapest University of Technology and Economics, Műegyetem rkp. 3, H-1111 Budapest, Hungary; bbodzay@mail.bme.hu; 4 Environmental Analytical and Geoanalytical Research Group, Szentágothai Research Centre, University of Pécs, Vasvári Pál str. 4., H-7622 Pécs, Hungary; istvan.sajo@gmail.com

**Keywords:** upgrading, recycled polyethylene-terephthalate, montmorillonite, flame retardancy, mechanical properties, prototype development

## Abstract

Recycled polyethylene-terephthalate (rPET) nanocomposites of reduced flammability were prepared by combining aluminum-alkylphosphinate (AlPi) flame retardant (FR) and natural montmorillonite (MMT), in order to demonstrate that durable, technical products can be produced from recycled materials. During the development of the material, by varying the FR content, the ratio and the type of MMTs, rheological, morphological, mechanical and flammability properties of the nanocomposites were comprehensively investigated. Related to the differences between the dispersion and nucleation effect of MMT and organo-modified MMT (oMMT) in rPET matrix, analyzed by Scanning Electron Microscopy (SEM), Energy Dispersive X-Ray Spectroscopy (EDS) and Differential Scanning Calorimetry (DSC), mechanical properties of the nanocomposites changed differently. The flexural strength and modulus were increased more significantly by adding untreated MMT than by the oMMT, however the impact strength was decreased by both types of nanofillers. The use of different type of MMTs resulted in contradictory flammability test result; time-to-ignition (*TTI*) during cone calorimeter tests decreased when oMMT was added to the rPET, however MMT addition resulted in an increase of the *TTI* also when combined with 4% FR. The limiting oxygen index (LOI) of the oMMT containing composites decreased independently from the FR content, however, the MMT increased it noticeably. V0 classification according to the UL-94 standard was achieved with as low as 4% FR and 1% MMT content. The applicability of the upgraded recycled material was proved by a pilot experiment, where large-scale electronic parts were produced by injection molding and characterized with respect to the commercially available counterparts.

## 1. Introduction

The PET usage as a packaging material increases year by year, and therefore the PET bottle waste also expands rapidly due to the fact that the life of a plastic bottle is brief [1,2]. The recycling of the PET waste is an important environmental question and the answer could be the upgrading recycling of flakes to technological plastics even if the longer lifetime is required by the potential application fields. It is claimed that morphological and mechanical properties of polyesters are just slightly decreasing with recycling when the optimal technology is applied [3,4], however the degradation which is characterized by the value of intrinsic viscosity (IV) could increase the crystallinity and the modulus of the material and decrease the impact strength [5,6]. Nevertheless, there is always an opportunity to improve these properties, e.g., by using fiber reinforcement, blending with other polymers or adding some extra additives [7,8,9,10]. Quality improvement could be achieved in other aspects of the polymer as well, such as by increasing its fire retardancy [11,12,13] and thus open the possibility for new applications, such as in electrical and electronic products.

Nowadays, it is a global industrial problem that an increasing number of common fire retardants that proved to be effective even in smaller doses, such as systems containing halogen, are now forbidden or under a process of restriction. The presently used alternative solutions, such as metal-oxides, metal-hydroxides, phosphorous compounds etc. are usually expensive, and need to be used in larger quantities that make them even more costly. Moreover, they often cause deterioration of the mechanical properties [14,15,16,17,18]. The researchers strive to develop fire retardants that can satisfy the standards but do not raise the price of the products significantly and do not decrease the mechanical properties of the polymer, in ideal situations they even improve it [19,20].

In the case of halogen-free aluminum-alkylphosphinate (AlPi), the dominant flame retardant mechanism is through the release of phosphinate compounds that inhibit the chemistry of the gas-phase combustion and the increase of the carbonaceous residue (char) production that invoke a thermal barrier effect [21]. Besides, it changes the melt viscosity of the matrix polymer and therefore increases the dripping behavior [22,23].

The flame retardant effect of nanostructured materials is widely investigated. Adding carbon nanotubes (CNT) [24,25], montmorillonite (MMT) [26,27], boehmite [28] or sepiolite [29] the flammability of polyesters can be reduced, although using these substances alone, high level of flame retardancy (e.g., V0 classification according to UL-94 standard) cannot be achieved. The use of MMT as a synergic additive to AlPi for designing polyesters with better flame retardant properties has already been studied by researchers, however, there are some contradictory results in the literature about the required quantities and the necessity and nature of the surface treatments.

Ye et al. [30] analyzed poly(lactic acid) (PLA) with AlPi and natural MMT modified with methyl-tallow-bis(2-hydroxyethyl) ammonium. Authors concluded that the dominant reaction was the char formation and melt dripping disappeared when either FR or oMMT was added to the matrix. V0 classification according to the UL-94 standard was only achieved when 17% AlPi and 3% oMMT were used together.

Kim et al. [31] investigated the effects of MMT modified with phosphonium salt of dodecyltriphenyl in in situ polymerized poly(butylene terephthalate) (PBT) matrix. According to the authors, the initial decomposition temperature increased slightly when 1–2% of modified MMT was used.

Ramani et al. [23] concluded that there is a synergetic effect when 2.5% quaternary ammonium salt modified MMT (oMMT) and 15.5% AlPi was added to glass fiber reinforced PBT (GF-PBT). Due to the addition of 2.5% oMMT to the flame retardant composite, the limiting oxygen index (LOI) value increased from 31.5 to 35.5%. In this study, it was established that the presence of AlPi led to char formation while adding oMMT led to the formation of inorganic deposits that increased the viscosity of the GF-PBT. Based on cone-calorimetry using different external heat fluxes, it was shown that AlPi flame retardant with oMMT was more resistant to ignition than the rest of the materials at the lower heat flux (22.5 kW/m^2^). This was explained by the water content in the crystal lattice, which induces the hydrolytical decomposition of AlPi to produce phosphorus containing radicals. This could increase the *TTI* through radical scavenging mechanisms. During decomposition of MMT, water and carbon dioxide gases were produced which diluted the decomposing olefinic compounds emerging from the disruption of the polyester matrix. Based on the authors experiment at a higher external heat flux (30–90 kW/m^2^) the crystalline water escapes before reacting with AlPi and at the same time the phosphorous radical species also escape the flame front. Hence the *TTI* is shorter when compared with PBT containing only flame retardant.

Louisy et al. [32] tested similar FR compositions in GF-PBT matrix. 20% AlPi and 18% AlPi + 2% MMT modified with quaternary ammonium salt were added. Composite with oMMT content showed slightly lower LOI value compared to the composite containing only AlPi (39% to 40%).

Ge et al. [33] investigated the effect of oMMT (organically modified by octadecyltrimethyl-ammonium chloride) on PET–2-carboxyethyl(phenylphosphinic) acid (PET-*co*-HPPPA) copolymer. HPPPA content was 5% and the ratio of oMMT was varied between 1–3%. LOI index shifted from 31.5% to 34% by adding 1% of oMMT and it did not change after any further increase of oMMT content. UL-94 results show V2 rating at 0 and 1%, and V0 at 2 and 3% oMMT content.

Habibi et al. [34] prepared PET-oMMT nanocomposites with 0, 3 and 5% oMMT content. Based on their cone-calorimeter tests it was found that flame retardant properties of nanocomposites improved with increasing clay content. The nanocomposite containing 5% oMMT showed adequate flame retardancy and dripping resistance. Besides, decreasing LOI values of the PET/oMMT composites ware measured with increasing oMMT content.

The reason for developing PET-MMT systems is primarily not only the flame retardancy of the material but the ability to improve the gas barrier and mechanical properties [35,36,37,38,39]. The role of surface modification in the development of properties is an intensively researched area.

Pegoretti et al. [40] used montmorillonites without modification and ion-exchanged MMT modified by quaternary ammonium salts and added to regranulated PET flakes. By analyzing the nanocomposites’ mechanical and morphological properties they established that the composites containing modified MMT were able to form an intercalated structure. However, only a small interlayer space shift was observed when unmodified MMT was added to the PET matrix, indicating weak intercalation.

Wang et al. [41] researched original PET nanocomposites with added organo-modified MMT. The interlayer spacing of the organo-modified MMT increased which was explained by intercalation. By analyzing the mechanical properties, they found that by adding 1 wt % MMT to the system the yield stress and flexural strength improved, but when 3 or 5 wt % were added the mechanical properties deteriorated. These findings can be explained by the increasing quantity of the filler, which resulted in the decrease of dispersion and lower intercalation degree. The oMMT increased the heat deflection temperature (HDT) of the PET as a function of increasing filler content, however the impact strength decreased, which was explained by the decreasing moving ability of the molecules.

Kracalik et al. [42] prepared recycled PET (rPET) composites using 5% clay in a twin-screw extruder and then compared the dispersion of the different types of MMTs. They evinced that by raising the polarity of the surface better delamination can be achieved, and by mixing with the polar PET intercalation is also possible. Decomposition of alkylammonium ethers of the organo-modifier influenced the PET degradation during the processing.

Zare summarized in his review article [13] the benefits of using MMT in recycled polymers. The author showed that substantial increase of the modulus can be achieved by high MMT content, although the optimum strength and stiffness is around 2% MMT content, due to the fact that the mobility of the molecular chains is influenced by the nanofiller.

Vassiliou et al. [43] prepared organo-modified MMT nanocomposites in in situ polymerization of PET. With these well dispersed nanoparticles substantial improvement of the strength of the composites was achieved.

In summary, there are contradictory results in the literature regarding the dispersibility of neat and organo-modified MMTs in polyester matrix materials, furthermore the mechanical properties of the nanocomposites are barely studied, especially when flame retardant compositions are investigated (Table 1).

The aim of this study is the upgrading recycling of rPET by preparing flame retarded nanocomposites accompanied with adequate flammability and mechanical properties at the same time. V0 rating according to the UL-94 standard with reduced FR content was intended to be reached by optimizing the type and ratio of nanoclay type synergist. From the developed recycled material prototype of an electrical product (TV cover) was manufactured by injection molding and comprehensively characterized to demonstrate the feasibility of preparation of durable products from recycled raw materials.

## 2. Materials and Methods

rPET flakes (Jász-Plasztik Kft, Jászberény Hungary), originating from collected, washed and sorted post-consumer PET bottles, with an intrinsic viscosity (IV) value of 0.70 dL/g was used as matrix material. The average PE and PVC content of the rPET flakes was measured to be 25 and 20 ppm, respectively.

Exolit OP 1240 (Clariant, Muttenz, Switzerland) aluminum-tris-(diethylphosphinate) with a phosphorus content of 23.3–24.0% was used as flame retardant (FR) additive.

Cloisite 116 (Byk, Wesel, Germany) natural montmorillonite (MMT) and Cloisite 5 (Byk, Germany) natural montmorillonite modified with bis(hydrogenated tallow alkyl)dimethyl salt (oMMT) were used as nanofillers.

The rPET flakes were dried for 4 h at 150 °C, and then mixed together with the additives. LT 26-44 (Labtech Engineering, Samut Prakan, Thailand) twin screw extruder was used for the mixing with a melt temperature of 265 °C. The produced regranulate was dried for another 4 h at 150 °C, then 80 mm × 80 mm × 2 mm specimens were prepared by Allrounder Advance 370S 700-290 (Arburg, Lossburg, Germany) injection molding machine, with the following parameters: Melt temperature: 270 °C, maximum injection pressure: 900 bar, mold temperature: 60 °C. Table 2 shows the composition of the test samples prepared for the optimization of the composition of the recycled product.

The interlayer spacing was measured in MMT and oMMT with wide-angle X-ray diffraction (WAXD). WAXD analysis was performed by PW 3710 (Philips, Amsterdam, the Nederland) based PW 1050 Bragg-Brentanopara focusing goniometer using CuKα radiation (λ = 0.15418 nm).

Thermogravimetric analysis (TGA) measurements were carried out on the used additives and prepared nanocomposites using a Labsys Evo (Setaram, Caluire-et-Cuire, France) instrument with a heating rate of 20 °C/min under nitrogen gas flow, covering a temperature range of 50–800 °C. About 6–8 mg of sample was used in each test.

SEM micrographs were obtained from the cryogenic fracture surfaces of the nanocomposites using EVO MA 10 instrument (Zeiss, Oberkochen, Germany) with an accelerating voltage of 30 kV. The samples were coated with 32 nm gold layer before examination in order to prevent charge build-up on the surface. The dispersion of the additives was investigated via energy dispersive X-ray spectrometry (EDS) using an Octane Pro type (AMATEX EDAX, Mahwah, NJ, USA) apparatus. In this case the thickness of the gold coating was 5 nm. Element mapping was carried out with an accelerating voltage of 15 keV and an amplification of 500×.

Mass loss type cone calorimeter tests were carried out by an instrument delivered by Fire Testing Technology Ltd. (East Grinstead, UK) based on the ISO 5660-1 standard method. 2 stacked pieces of injection molded specimens with dimensions of 80 mm × 80 mm × 2 mm were exposed to a constant heat flux of 50 kW/m^2^ and ignited. Heat release values and mass reduction were continuously recorded during burning. The average effective heat of combustion (*AEHC*) [MJ/kg] was calculated according to Equation (1), where *HRR* [kW/m^2^] is the heat release rate per unit exposed area, *Δt* is the sampling time interval (in this case 1 s), *TTI* is time to ignition, *EOF* is time to end of flame and *m* [kg/m^2^] is the mass of specimen per unit exposed area. The fire performance index (*FPI*) [sm^2^/kW], a useful parameter that can be calculated as the ratio between the time to ignition (*TTI*) [s] and the peak of heat release rate (*HRR_max_*) [kW/m^2^], was calculated according to Equation (2).

(1) $$AEHC=\;\frac{\sum_{TTI}^{EOF}HRR*\Delta t}{m_{TTI}-m_{EOF}}$$

(2) $$FPI=\;\frac{TTI}{HRR_{max}}.$$

The *FPI* value gives important information about the degree of fire hazard [44,45].

The flame retardant performance of the prepared samples was characterized by limiting oxygen index (LOI) measurements according to the ASTM D 2863 standard. The LOI value expresses the lowest oxygen to nitrogen ratio where specimen combustion is still self-supporting.

Standard UL-94 tests were performed in a UL-94 chamber (Wazau, Berlin, Germany) with methane gas. Specimen thickness was 2 mm. UL-94 classification is used to determine dripping and flame spreading rates. First, horizontal burning tests were carried out. As long as the burning rate did not exceed 75 mm/min over a 75 mm span, the specimen got HB classification. If the burning stopped before it reached the 25 mm mark on the specimen, then the vertical burning test was carried out as well.

Three-point-bending tests were carried out using Z020 type (Zwick, Ulm, Germany) universal testing instrument (Zwick, Ulm, Germany) at room temperature. The test speed was 5 mm/min, support span was 64 mm.

Impact tests were carried out by Resil Impactor Junior (Ceast, Pianezza, Italy), using notched specimens. The measurements were performed at room temperature with a pendulum of 2 J and with a velocity of 2.9 m/s.

The morphological characteristics of RPET injection molded specimens were determined with a TA Q2000 type (TA Instruments, USA) DSC device at a heating rate of 10 °C/min under 25 mL/min nitrogen gas flow, covering the temperature range of 20 and 290 °C (one heating cycle). The weight of the examined samples was between 6 and 8 mg. Crystalline fraction (*CRF*) was calculated by equation (Equation (3)):

(3) $$CRF=(\left(\Delta h_{m}-\sum \Delta h_{cc}\right)/\left(\Delta h_{m}^{0}-\left(1-\alpha \right)\right)100\%,$$

where *CRF* is crystalline fraction in the sample [%], *Δh_m_* is the specific enthalpy of melting [J/g], *Δh_cc_* is the specific enthalpy of cold crystallization [J/g], *Δh_m_*^0^ is the specific melting enthalpy of 100% crystalline PET (140.1 J/g) and *α* is the ratio of additives [46].

The intrinsic viscosity (IV) of the PET material and the specimens was determined using a computer controlled PSL Rheotek automatic solution viscometer equipped with an optical sensor. Phenol-tetrachloroethane mixture in the ratio of 60/40 was applied as a solvent—the concentration was 0.5 g/dL, and examination temperature was 30 °C.

## 3. Results

### 3.1. Composition Optimisation

#### 3.1.1. Characterization of Nanoclays and Nanocomposites

**Figure 1:**
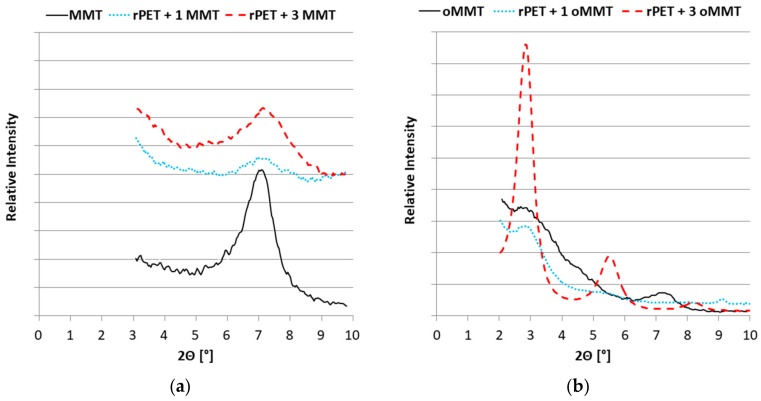
Interlayer spacing of MMT (**a**) and oMMT (**b**).

The interlayer spacing of the MMT and the oMMT were measured before and after processing. Figure 1 shows that the diffraction angle did not change significantly either using the MMT nor the oMMT. According to the Bragg law (Equation (4)), the interlayer spacing of MMT and oMMT for the first diffraction is:

d = λ/(2sinθ),(4)

where d is the interlayer spacing [nm]; λ is the wavelength [nm] and θ is diffraction angle [°].

The results are in accordance with the basal interlayer spacing (Table 3) [47]. The slight decrease in the case of the 3% oMMT might be due to the dehydration of layered silicate during the drying process [48] or the degradation of the organo-modifier during the high-temperature processing. The WAXD results do not indicate exfoliation or the development of intercalated structure for either of the examined nanoclay.

**Figure 2:**
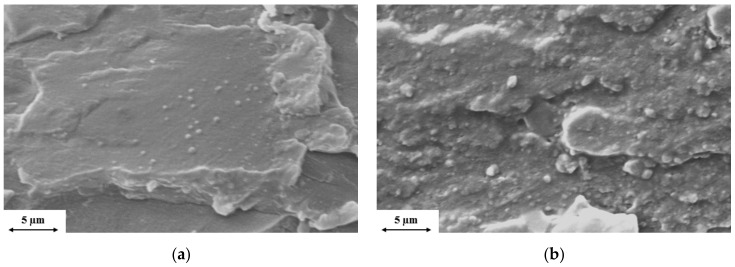
SEM images of fracture surface of (**a**) 97% rPET + 3% MMT and (**b**) 97% rPET + 3% oMMT.

In the SEM pictures of Figure 2, the cryogenic fracture surface of the nanocomposites with 3% clay content can be seen. MMT shows finer dispersion (Figure 2a) than oMMT (Figure 2b) where more aggregated structures can be observed.

**Figure 3:**
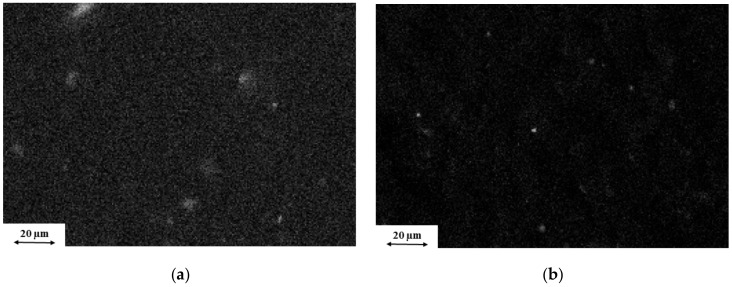
EDS images of fracture surfaces (white dots mark silicon elements) of (**a**) 97% rPET + 3% MMT and (**b**) 97% rPET + 3% oMMT.

The two EDS pictures in Figure 3 confirms this assumption. The dispersion of silicon element, which is a specific component of nanoclay, in the rPET matrix is finer in the case of the MMT and much coarser when oMMT was added.

**Figure 4:**
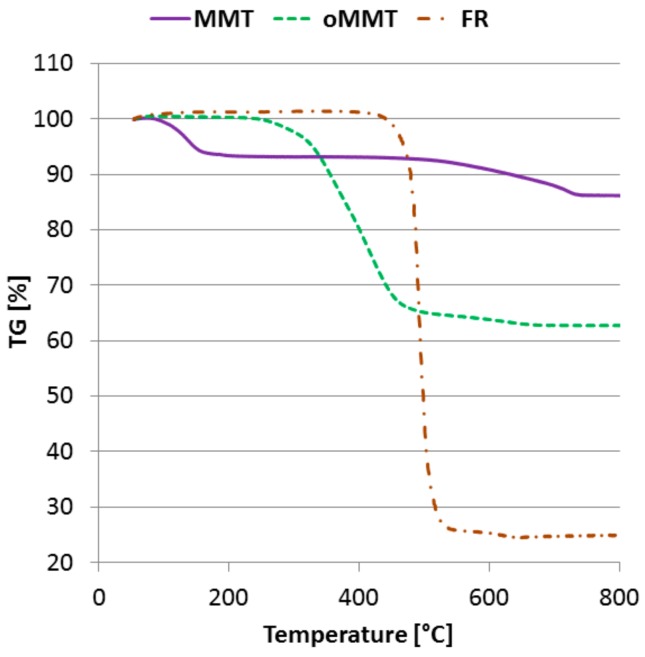
Thermogravimetric curves of the additives (N_2_ atmosphere, 20 °C/min).

Based on thermogravimetric analyses of the additives (Figure 4), the used AlPi decomposes rapidly around 500 °C and loses 75% of its weight. The thermal degradation of neat MMT occurs in two steps; 7% weight loss can be measured both between 80–150 °C and between 500–750 °C. The first step is due to the evaporation of water, while the second is caused by the dehydroxylation of MMT [49]. In the case of oMMT the weight loss is not significant until 250 °C (<2%). In the 250–460 °C interval the organo-modifier decomposes. This confirms that during compounding and injection moulding (270 °C) decomposition of the organo-modifier of oMMT can occur. Between 500–700 °C the additional weight loss is similar to the one observed in the MMT’s curve.

**Figure 5:**
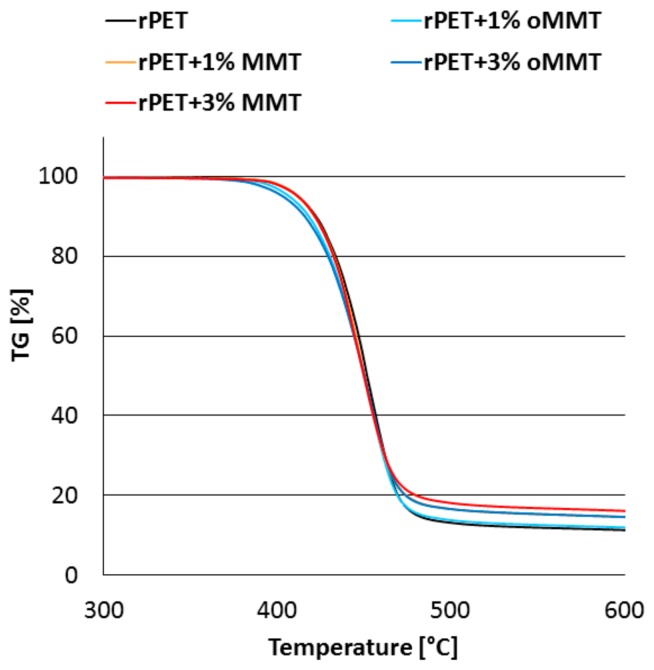
Thermogravimetric curves of the nanocomposites (N_2_ atmosphere, 20 °C/min).

The early thermal decomposition of the organo-modifier of oMMT is also observable on the TGA curves of the prepared nanocomposites (Figure 5), it decreases the initial thermal degradation temperature of the oMMT containing rPET samples. When considering the amount of residue obtained at 600 °C (Table 4), it can be found that despite the identical nanoclay contents, a higher amount of char remained from the un-treated MMT containing rPET samples, indicating char promoting behavior of natural MMT.

**Figure 6:**
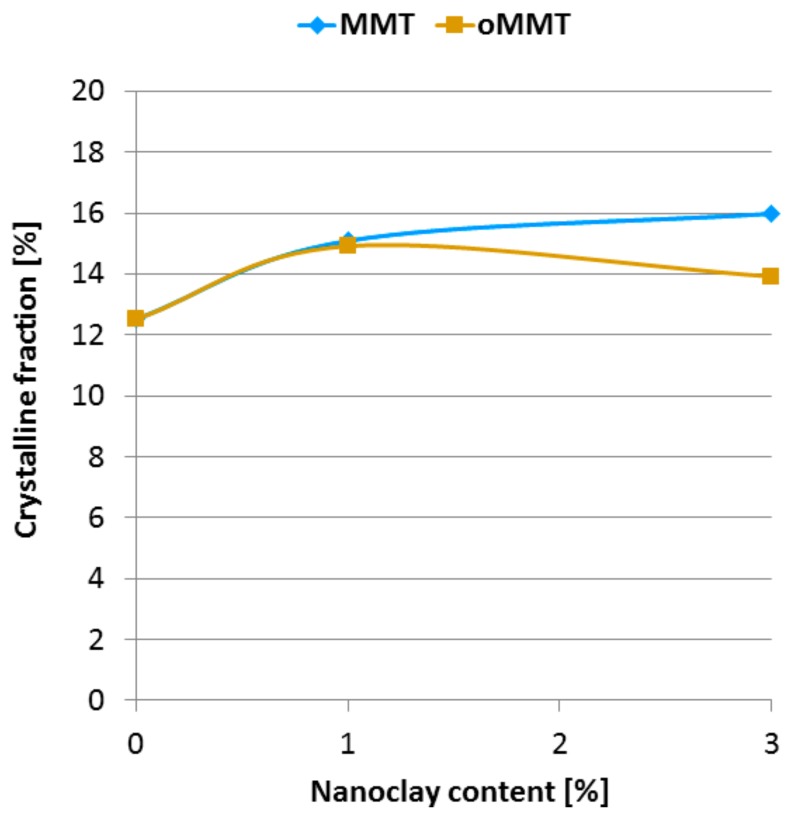
Crystalline fraction of the nanocomposites as a function of nanoclay content.

The crystalline fraction of the nanocomposites containing MMT and oMMT was calculated based on DSC curves according to Equation (2). (The DSC curves can be found in the Figure S1) It can be seen in Figure 6. that both the MMT and the oMMT slightly increased the crystalline fraction of the rPET. This increase can be explained by the nucleating effect of the nanoclays. The neat MMT seems to increase the crystalline content of rPET more effectively than oMMT, indicating better dispersion of the MMT nanoparticles, as also found based on SEM and EDS analyses. Furthermore, the molecules certainly degraded during the high-temperature processing, i.e., the length of the chains got reduced and thus their movements were less hindered, which also results in an easier organization of the chains [41].

#### 3.1.2. Flammability of rPET Nanocomposites

**Figure 7:**
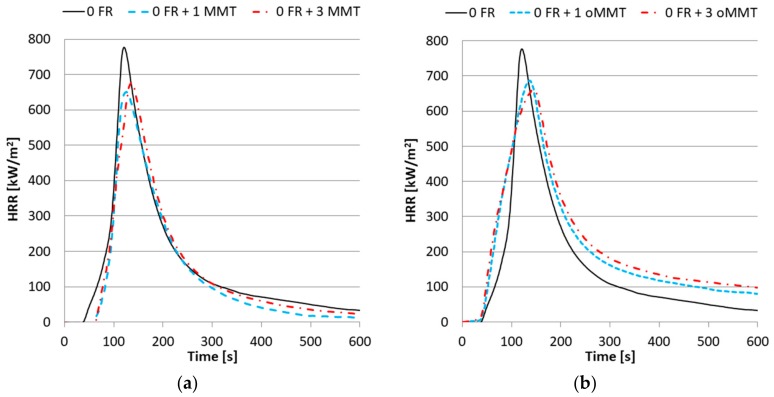
Heat Release Rate curves of nanocomposites with (**a**) MMT filler; (**b**) oMMT filler.

The peak of heat release rate (*HRR_max_*) and its appearance (*HRR_max_* Time), time-to-ignition (*TTI*), total heat release (*THR*), fire performance index (*FPI*), average effective heat of combustion (*AEHC*) and residual mass values were determined from cone calorimetry. The *HRR_max_* of samples without FR changed similarly when oMMT or MMT was added to the system (Figure 7). The oMMT content did not influence the *TTI*, however the MMT increased it by 20-22 s (Table 5). Surprisingly, the *THR* and *AEHC* were measured to be increased by the oMMT. In theory, nanoclays should not change the *THR* as they do not act in the vapor phase, their main flame retardant effect, the barrier/insulation effect, is expected to reduce the *HRR_max_* values. The observed increase of *THR* with oMMT is proposed to be related to the viscosity relations. oMMT addition increases the viscosity of the rPET, thereby inhibiting the dripping and promoting the combustion. This effect would offset the barrier effect of the nanoclay partially.

**Figure 8:**
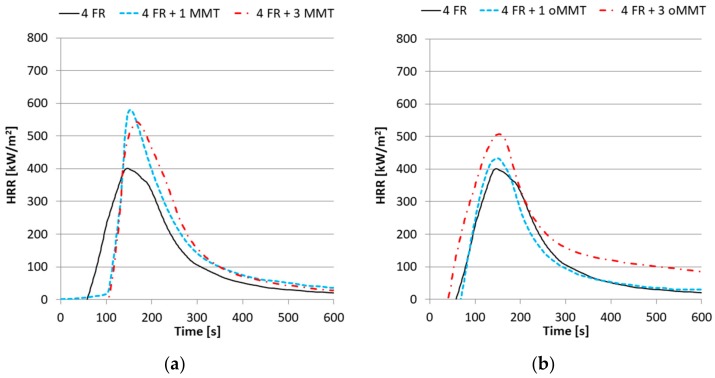
Heat Release Rate curves of nanocomposites with 4% FR and (**a**) MMT filler; (**b**) oMMT filler.

The *HRR_max_* of the rPET samples decreased nearly by 50% and the *TTI* increased when 4% FR was added (Figure 8). The *TTI* increased slightly when the sample contained 1% oMMT but decreased when it contained 3%. When MMT was used either by 1 or 3% the *TTI* increased identically by 44–46 s. The *HRR_max_* increased parallel with the oMMT content. In contrast, when 1% MMT was added to the system the *THR* did not change notably whereas 3% MMT content resulted in an increase of the *THR*.

**Figure 9:**
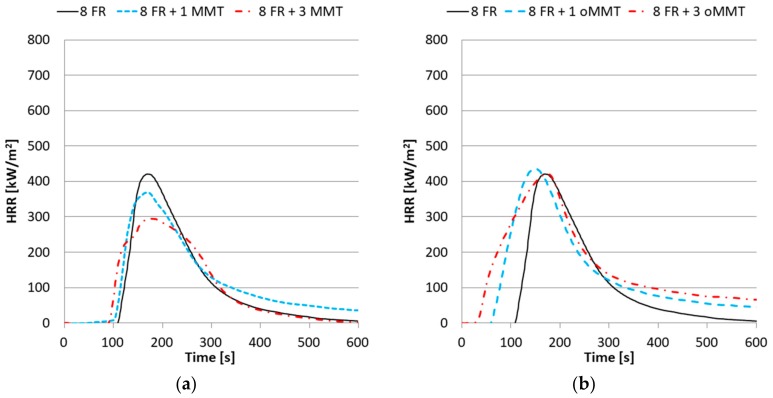
Heat Release Rate curves of nanocomposites with 8% FR (**a**) and MMT filler; (**b**) oMMT filler.

Further increase of the FR content did not affect the peaks of *HRR* considerably; however, the *TTI* was increased significantly. The samples containing 8% FR acted slightly different when compared to the samples containing 0 or 4% FR (Figure 9). The difference between these samples is that by adding MMT to the system the peaks of *HRR* (*HRR_max_*) decreased even further, however the *TTI* decreased only slightly. In contrast, the oMMT significantly reduced the *TTI* value at both percentages.

Regarding the charring ability of the samples, indicated by the residual mass values obtained after cone calorimetry (Table 5), noticeable beneficial effect of untreated MMT addition was found, while oMMT did not show any influence in this respect. As much as 15% char remained from the 4% FR + 1% MMT containing the sample.

Table 5 clearly shows that without adding FR, oMMT does not noticeably influence the *FPI* value, while MMT increases it to its two-fold. Similarly, when the nanoclays are used in combination with 4 or 8% FR, oMMT causes reduction of the FPI, while MMT increases it further.

As it is presented in Table 5, UL-94 rating of rPET is HB while adding 4 or 8% FR V2 level can be achieved. oMMT had no effect on the UL-94 rating, however MMT raised the classification from HB to V2 at 0% FR content, and from V2 to V0 at 4 and 8% FR content, respectively. Due to the synergetic effect between the AlPi type FR and natural MMT, V0 rating was reached with as low as 5% of additives (4% FR + 1% MMT), which is much less then published before in the literature [30]. Based on the low concentration of additives necessitated to reach an adequate level of flame retardancy in rPET, less deterioration of the mechanical properties of the proposed recycled products was expected.

**Figure 10:**
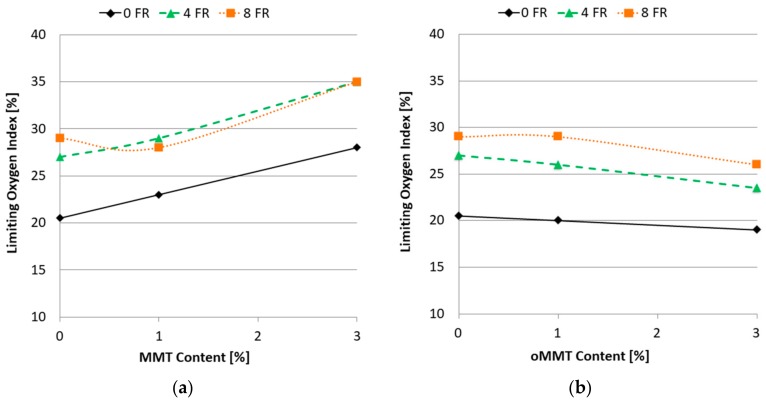
Results of limiting oxygen index (LOI) tests as a function of filler content: (**a**) MMT filler; (**b**) oMMT filler.

As it is shown in Figure 10, LOI values increased by raising the FR concentration in the rPET matrix. The different effect of the two types of montmorillonites appeared here as well. The MMT addition in general further increased the oxygen index value, at as low as 5% additive content (4% FR + 1% MMT) an LOI of 29% was reached. In contrast, similarly to the finding of Louisy et al. [32], oMMT addition slightly lowered the LOI values.

Based on the flammability test results it was concluded that the heat barrier and char promoting behavior of nanoclays can only prevail when adequate dispersion is achieved in the polymer matrix. Accordingly, in our case the neat MMT at low concentration (1%) showed the best flame retardant performance, especially when combined with 4% AlPi type FR.

#### 3.1.3. Mechanical Properties of rPET Nanocomposites

**Figure 11:**
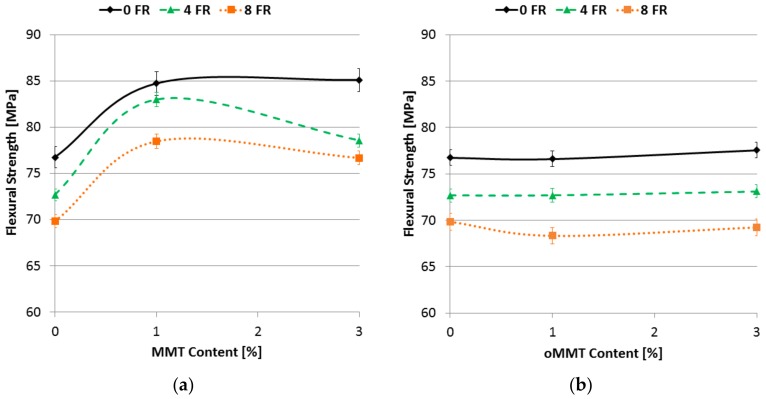
Flexural strength as a function of filler content: (**a**) MMT filler; (**b**) oMMT filler.

The flexural strength decreased with increasing FR content (Figure 11). However, the extent of this decrease was minor, with 4% FR content only slightly more than 5% decrease can be seen and with 8% FR content the decrease was 9%. 1% MMT content increased the flexural strength of the specimens by 10%, however further increase of the MMT content did not change it significantly, moreover with the specimen containing 4% FR even some fallback can be seen in Figure 11a. The flexural strength did not effectively change with the oMMT addition (Figure 11b).

**Figure 12:**
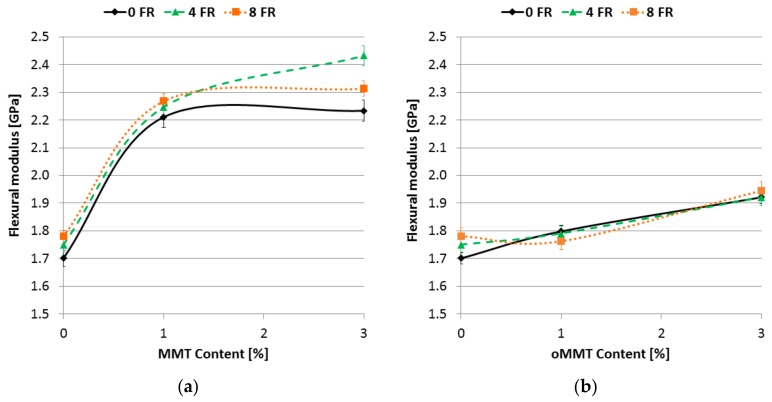
Flexural modulus as a function of filler content: (**a**) MMT filler; (**b**) oMMT filler.

The flexural modulus increased with raising nanofiller ratio independently from the type of the MMT (Figure 12), although the extent of this increase is not the same. By adding 1% MMT to the matrix the modulus increased by more than 30% independently from the FR content. The effect of oMMT addition is less pronounced, up to 10% increase in flexural modulus was measured when 3% oMMT was added. Based on this phenomenon, a conclusion can be drawn that the static mechanical properties are mostly influenced by the dispersion degree of the nanoclays, and not by the indirect increase of the formed crystalline fraction. The amount of crystalline fraction was similar when 1 % MMT or oMMT was used, however the increase in flexural strength and modulus differs.

**Figure 13:**
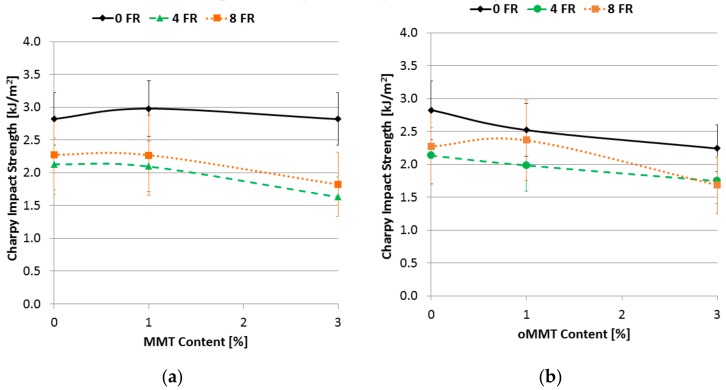
Charpy impact strength (notched) as a function of filler content (**a**) MMT filler; (**b**) oMMT filler.

Figure 13 shows the notched Charpy impact strength of the samples. In the case of samples without FR the MMT did not modify the impact strength considerably. In the case of the FR containing samples the impact strength did not change notably when the MMT content was 1%, however by raising the MMT ratio to 3% the impact strength slightly decreased. The main reason for this is that by raising the MMT content the dispersion of the nanoclay decreased in the matrix. Much of the available literature [39,43] notes that by using organo-modified MMT finer dispersion can be achieved that results in increased impact strength properties or only slighter decrease, compared to the ones obtained with untreated MMT. In contrast to this statement by adding oMMT to the rPET the specific impact strength did not change as it was predicted. In samples without FR the impact strength decreased linearly with the raising oMMT content; with 1% oMMT content the decrease was about 11%, and with 3% of oMMT it was about 20%. The impact strength of the samples containing 4% FR did not change significantly with 1% oMMT content however when the oMMT ratio reached 3% the impact strength decreased even by 20%; this value is similar to the one measured with MMT. This suggests that the organo-modifier was not able to exert its effect. It can be clearly seen in Figure 13 that there is a steady decline in the impact strength when the FR content is 8%. At 3% oMMT content the impact strength already decreased by 25%.

### 3.2. Pilot Experiment

When the mechanical properties were compared of the produced V0 classified recycled materials, the best results were achieved by the following recipe: rPET + 4% FR + 1% MMT. This material proved to be suitable for the production of television parts by injection molding. The properties of the developed recycled material were compared to alternative materials used in the E&E industry. According to Table 6, the strength and modulus of the developed recycled material are at a comparable level with the strength and modulus of the alternative materials like PC/ABS. Furthermore, these mechanical properties of the recycled PET bottles far exceed the properties of a fire resistant HIPS material. The technical data sheets of the alternative materials do not contain the unnotched Charpy impact strength of the materials; therefore, the measured 20.6 kJ/m^2^ impact strength of the developed rPET samples cannot be compared with them.

**Figure 14:**
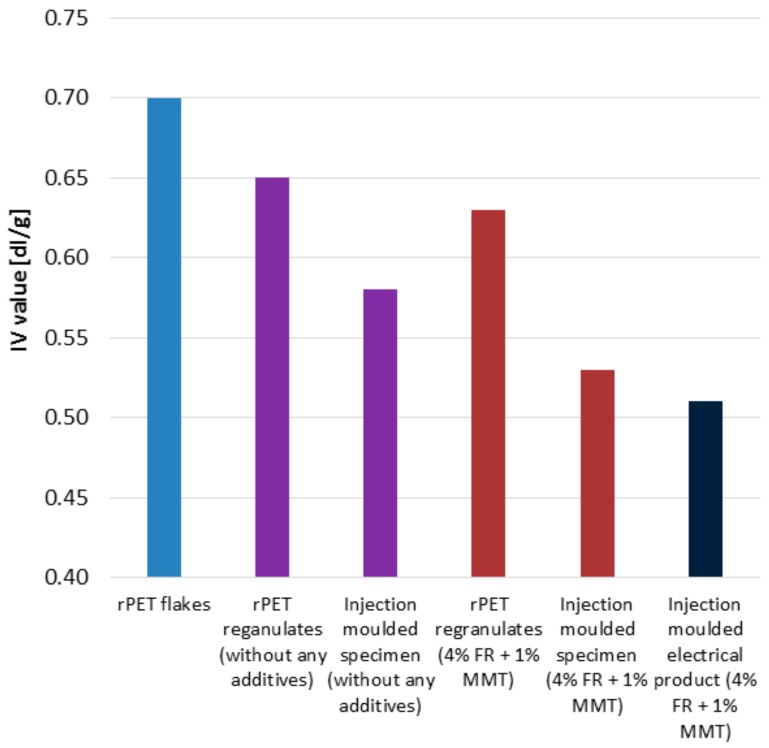
Change in the IV value during reprocessing with- and without additives.

The impact resistance of the rPET with a v-notch falls behind the other materials used in television parts. This means that the prepared material is sensitive to cracks. When the product is being designed, this has to be taken into consideration. The low impact strength is mainly due to the hydrolytic degradation of the PET caused by the presence of water during the processing. The degradation of the rPET can be traced by monitoring the IV values as presented in Figure 14. The IV value of the initial PET flake is 0.70 dL/g. In the case of making regranulates without any additives, the IV value decreases to 0.65 dL/g due to the extrusion process. Further processing these regranulates by injection molding decreased the IV value even further and reached the value of 0.58 dL/g. It can be seen that the whole technological process decreased the IV by 0.12 dL/g. The IV value decreased more significantly when 4% FR and 1 % MMT was mixed with the PET flakes. By considering all the processing steps, the IV value decreased by 0.17 dL/g, after the extrusion it was 0.63 dL/g and the following injection molding process decreased it to 0.53 dL/g. It can be concluded that the technology and the additives are both responsible for the degradation of the polymer. The insufficient notched impact strength is certainly associated with the low IV value of the recycled nanocomposite product.

The pilot experiment was carried out with an ENGEL Duo 11500 type injection molding machine with a hot runner and with one sprue. The developed material containing rPET + 4%FR + 1%MMT was used for the production of 0.9 kg TV back covers. The adjusted parameters are:

Zone temperatures: 270–285 °C; Hot runner temperature: 275 °C; Mold temperature: 70 °C; Injection time: 4.2 s; Holding time: 8.0 s; Remaining cooling time: 35 s; Injection speed: 30 mm/s; Injection pressure: 1700 bar; Holding pressure: 800 bar; Back pressure: 100 bar.

Specimens were cut out from the injection molded products for mechanical tests. The Charpy impact resistance was 2.0 kJ/m^2^ on the notched specimens and 17 kJ/m^2^ on the unnotched specimens. The slight difference in values from the one measured on the standardized specimens and the specimens cut from the product can be explained with the degradation differences that occur because of the different processing technologies. (The hot runner mold could cause more shear- and thermal degradation on the polymer; higher melt temperature was needed for the proper filling of the mold.)

The measured IV value of 0.51 dL/g of the specimen cut out from the product supports this assumption. Due to the sensitivity of the material to the technology the PET and the additives should be carefully dried; and it is extremely important that the right processing machines and technological parameters should be chosen. To compensate for the degradation and by that increase the impact resistance of the material chain extender additives could be used, or the material could be treated in a solid state polymerization (SSP) reactor [50].

## 4. Conclusions

In this work, the type (untreated or organomodified) and amount (1 to 3%) of montmorillonite type nanosynergists were investigated on the flammability and mechanical performance of recycled PET flame retarded with aluminum-alkylphosphinate with the aim to find the best flame retardant composition for production of a technical product from recycled PET.

The cone calorimetric measurements showed that by combining the FR with untreated MMT the heat release rate of the rPET composites was noticeably moderated. Due to their synergetic effect, when 4% metal-phosphinate and 1 or 3 % MMT were used, the ignition time significantly increased. Based on *HRR_max_* values better results were obtained with 1% nanoclay content than with 3% which can be explained by the difference in the dispersion. This assumption was confirmed by SEM and EDS measurements. By using oMMT the *HRR* maximum was only slightly moderated, and the *TTI* did not vary noticeably, either.

The flexural strength of the rPET composites decreased by using FR, however the extent of the decrease did not reach 10%. This value is among the best results that have been achieved nowadays. Both of the layered silicates resulted in an increase of the flexural modulus, accompanied by a decrease of the impact strength. A noticeable increase of the flexural strength was achieved with MMT addition, however by the addition of oMMT similar quality improvement could not be noticed. The distinct effect of the two kinds of nanofillers on the properties of the rPET composites can be traced back to the different degree of dispersion.

It was concluded that the combined application of AlPi and untreated MMT has several advantages; V0 rating according to the UL-94 standard is achievable with as low as 5% of additives (4% AlPi + 1% MMT) besides reaching an LOI value of 29%. Furthermore, improvement in flexural strength and modulus can be achieved without compromising the impact resistance of the flame retarded rPET composites.

The high-temperature processing and the additives caused degradation of the rPET macromolecules, which was traced by measuring the IV values by every processing steps. The reduced IV values are associated with reduced impact strength.

It was demonstrated that the developed recycled material, upgraded with flame retardancy and nanoclay type reinforcement, has comparable flame retardant performance and flexural properties as the polymers (PC/ABS, HIPS) that are currently widely applied in electrical parts. However, due to the unavoidable hydrolytic degradation of the macromolecules during reprocessing, the insufficient impact strength of the recycled material needs to be improved when considering its application in the electrical industry. Nevertheless, manufacturing of a television part was successfully accomplished by injection moulding of the rPET based material.

## Figures and Tables

**Table 1:** Literary summary of polyester/nanoclay composites: Dispersion and mechanical properties of neat and organo-modified montmorillonite (MMT) reinforcement.

Paper	Type of Polyester	Mechanical Properties	Nature of Nanoclay/Dispersion
Ye et al. [30]	PLA	Little increase in impact strength and tensile strength	oMMT: Mixed intercalated/exfoliated structures.
Kim et al. [31]	PBT	Increase in tensile strength	oMMT: Intercalation and clusters
Ramani et al. [23]	PBT	Not tested	oMMT: Not tested
Louisy et al. [32]	PBT	Not tested	oMMT: Not tested
Ge et al. [33]	PET–2-carboxyethyl (phenylphosphinic) acid (PET-*co*-HPPPA) copolymer	Not tested	oMMT: Strong intercalation
Habibi et al. [34]	PET	Not tested	oMMT: Intercalated morphology
Pegoretti et al. [40]	recycled PET	MMT and oMMT had no significant effect on tensile strength, elongation at break decreased, and modulus increased in both case	MMT: Weak intercalation oMMT: Strong intercalation
Wang et al. [41]	PET	Impact strength and elongation at break decrease in the function of oMMT	oMMT: Intercalation
Kracalik et al. [42]	recycled PET	Not tested	oMMT: Partial or no exfoliation
Vassiliou et al. [43]	PET	Increase in tensile strength	oMMT: Exfoliation

**Table 2:** Composition of the prepared samples.

	rPET [%]	FR [%]	MMT [%]	oMMT [%]
0 FR	100			
0 FR + 1 oMMT	99			1
0 FR + 3 oMMT	97			3
0 FR + 1 MMT	99		1	
0 FR + 3 MMT	97		3	
4 FR	96	4		
4 FR + 1 oMMT	95	4		1
4 FR + 3 oMMT	93	4		3
4 FR + 1 MMT	95	4	1	
4 FR + 3 MMT	93	4	3	
8 FR	92	8		
8 FR + 1 oMMT	91	8		1
8 FR + 3 oMMT	89	8		3
8 FR + 1 MMT	91	8	1	
8 FR + 3 MMT	89	8	3	

**Table 3:** Results of wide-angle X-ray diffraction (WAXD)S measurements.

	Diffraction Angle (2θ) [°]	Interlayer Spacing [nm]
MMT	7.07	1.25
rPET + 1% MMT	7.01	1.26
rPET + 3% MMT	7.01	1.26
oMMT	2.70	3.27
rPET + 1% oMMT	2.70	3.27
rPET + 3% oMMT	2.72	3.25

**Table 4:** Char amounts formed from thermogravimetric analysis (TGA).

	Char Amount [%]
rPET	11.3
rPET + 1% MMT	14.5
rPET + 3% MMT	16.2
rPET + 1% oMMT	11.9
rPET + 3% oMMT	14.5

**Table 5:** Results of cone calorimetry and UL-94 tests.

	*TTI* [s]	*HRR_max_* Time [s]	*HRR_max_* [kW/m^2^]	*THR* [MJ/m^2^]	*AEHC* [MJ/kg]	*FPI* [sm^2^/kW]	Residual Mass [%]	UL-94 Rating
0 FR	39	119	773	97	17.7	0.05	0	HB
0 FR + 1 oMMT	38	135	706	129	23.5	0.054	0	HB
0 FR + 3 oMMT	37	143	679	140	25.6	0.054	0	HB
0 FR + 1 MMT	61	123	649	80	14.6	0.094	2.7	V2
0 FR + 3 MMT	63	134	674	89	17.3	0.093	5.7	V2
4 FR	58	145	401	73	13.6	0.145	0.6	V2
4 FR + 1 oMMT	69	148	433	84	15.7	0.159	2	V2
4 FR + 3 oMMT	38	150	506	117	21.2	0.075	0.2	V2
4 FR + 1 MMT	104	151	579	71	15.2	0.18	14.8	V0
4 FR + 3 MMT	102	166	543	87	17.4	0.188	8.9	V0
8 FR	109	167	418	60	11.3	0.261	4.6	V2
8 FR + 1 oMMT	60	144	434	84	15.6	0.138	2.5	V2
8 FR + 3 oMMT	22	172	420	100	18.1	0.052	0	V2
8 FR + 1 MMT	93	164	367	71	14.9	0.253	15.9	V0
8 FR + 3 MMT	91	172	295	58	12.7	0.308	15.8	V0

**Table 6:** Comparison of raw materials used for TV parts.

	rPET+ 4 FR + 1 MMT	PC/ABS NH-1237*	HIPS VE-1801*
UL 94 rating [2 mm]	V0	V0	V0
Flexural strength[MPa]	83	85	32
Flexural modulus[GPa]	2.25	4.2	1.80
Charpy unnotched impact strength [kJ/m^2^]	20.6	no data	no data
Charpy notched impact strength[kJ/m^2^]	2.1	5.0	10.0

* http://www.lotteadms.com/jsp/eng/product_intro/sm_datasheet.jsp.

## Supplementary Materials

The following are available online at http://www.mdpi.com/2073-4360/11/2/233/s1, Figure S1: DSC curves of the rPET nanocomposites
