# Supplementary figures and images for: Development of Flame-Retarded Nanocomposites from Recycled PET Bottles for the Electronics Industry
Source: Polymers (Basel). 2019 Feb 1;11(2):233. doi: 10.3390/polym11020233 (PMC6419026; doi:10.3390/polym11020233)

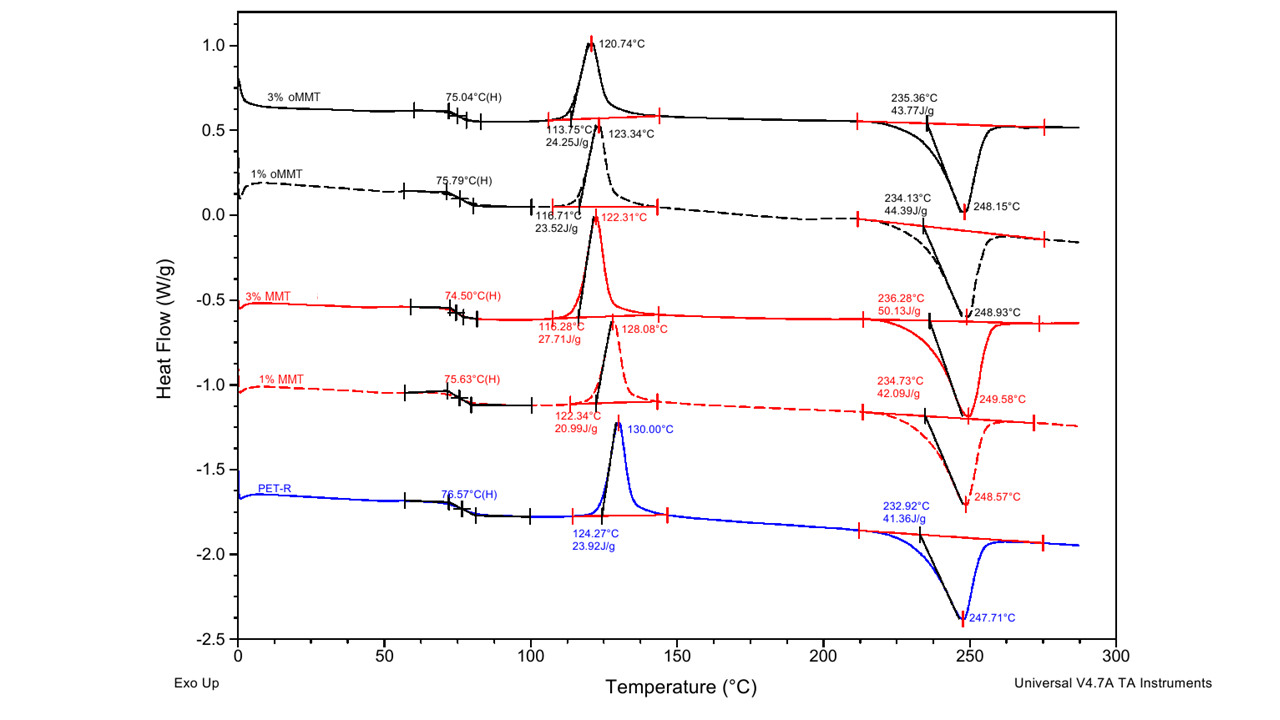

Supplement: Supplementary file 1 [file polymers-11-00233-s001.zip › polymers-427706-supplementary.tif]
